# Long-Term Survival of an Urban Fruit Bat Seropositive for Ebola and Lagos Bat Viruses

**DOI:** 10.1371/journal.pone.0011978

**Published:** 2010-08-04

**Authors:** David T. S. Hayman, Petra Emmerich, Meng Yu, Lin-Fa Wang, Richard Suu-Ire, Anthony R. Fooks, Andrew A. Cunningham, James L. N. Wood

**Affiliations:** 1 Cambridge Infectious Diseases Consortium, University of Cambridge, Cambridge, United Kingdom; 2 Institute of Zoology, Zoological Society of London, London, United Kingdom; 3 Rabies and Wildlife Zoonoses Group, Veterinary Laboratories Agency, Weybridge, United Kingdom; 4 Bernhard-Nocht Institute, Hamburg, Germany; 5 CSIRO Livestock Industries, Australian Animal Health Laboratory, Geelong, Australia; 6 Wildlife Division of the Ghana Forestry Commission, Accra, Ghana; 7 National Centre for Zoonoses Research, University of Liverpool, Liverpool, United Kingdom; Global Viral Forecasting Initiative, United States of America

## Abstract

*Ebolaviruses* (EBOV) (family *Filoviridae*) cause viral hemorrhagic fevers in humans and non-human primates when they spill over from their wildlife reservoir hosts with case fatality rates of up to 90%. Fruit bats may act as reservoirs of the *Filoviridae*. The migratory fruit bat, *Eidolon helvum*, is common across sub-Saharan Africa and lives in large colonies, often situated in cities. We screened sera from 262 *E. helvum* using indirect fluorescent tests for antibodies against EBOV subtype Zaire. We detected a seropositive bat from Accra, Ghana, and confirmed this using western blot analysis. The bat was also seropositive for Lagos bat virus, a *Lyssavirus*, by virus neutralization test. The bat was fitted with a radio transmitter and was last detected in Accra 13 months after release post-sampling, demonstrating long-term survival. Antibodies to filoviruses have not been previously demonstrated in *E. helvum*. Radio-telemetry data demonstrates long-term survival of an individual bat following exposure to viruses of families that can be highly pathogenic to other mammal species. Because *E. helvum* typically lives in large urban colonies and is a source of bushmeat in some regions, further studies should determine if this species forms a reservoir for EBOV from which spillover infections into the human population may occur.

## Introduction

*Marburgvirus* (MARV) and *Ebolaviruses* (EBOV) (family *Filoviridae*) can cause viral hemorrhagic fevers in humans and non-human primates when they spill over from their wildlife reservoir hosts [1], [2], [3], [4]. Disease outbreaks have case fatality rates in humans of up to 90%, depending on the viral type. Compelling evidence exists to suggest that some species of fruit bat act as reservoir hosts of the *Filoviridae* [1], [2], [3], [4], [5]. Towner et al. isolated virus and detected nucleic acids of genetically diverse MARV from the cave-dwelling fruit bat, *Rousettus aegyptiacus* and LeRoy et al. found serological and PCR evidence of EBOV infection in three other fruit bat species in West Africa [1], [4]. Subsequently, surveillance showed a higher seroprevalence against both MARV and EBOV in *R. aegyptiacus* than in other species tested [2]. Typically *R. aegyptiacus* live in very large roosts, with populations recorded over 100,000, which could facilitate persistence of infection within roosts.

The tree-roosting fruit bat, *Eidolon helvum*, is widespread and common across sub-Saharan Africa. It lives in large colonies, which sometimes number several million animals, often situated in cities [6], [7], [8], [9], [10]. The species is migratory, possibly in relation to food availability [8], [11]. In West Africa it was shown to migrate seasonally during the rainy season [8].

## Analysis

Ethical approval for this project (WLE/0467) was received from the Zoological Society of London Ethics Committee.

We screened sera from 262 *E. helvum* and 3 *Hypsignathus monstrosus* using indirect fluorescent tests for antibodies against EBOV subtype Zaire and MARV subtype Leiden [12]. Blood samples were collected from *E. helvum* in Ghana between January and April 2008 (n = 173) from urban colonies in Accra (n = 141) and Kumasi (n = 10) and from Tanoboase (n = 22) and from Accra (n = 89) in January and February 2009. Samples from 3 *H. monstrosus* were collected from Tanoboase in January 2009. The 262 *E. helvum* samples comprised 2 from neonate, 43 from sexually immature and 217 from mature bats, with an approxiate 2∶1 overall male bias. The 3 *H. monstrosus* were adult females. Bats were trapped on return to roosting sites in mist nets. Each bat in a subsample (n = 98) of the *E. helvum* population in Accra was fitted with a radio transmitter (Wildlife Materials Inc., Illinois, USA).

## Results

**Figure 1:**
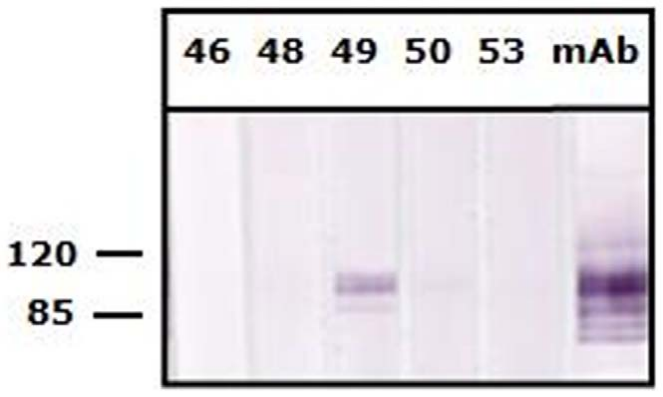
Western blot analysis using a recombinant nucleoprotein protein of EBOV-Zaire. The five bat sera (bat serum #46, 48, 49, 50 and 53, respectively) were tested at 1∶100. The positive control antibody (anti-RGS-His monoclonal antibody, Invitrogen, USA) in strip 6 was tested at 1∶600. The numbers on the left are molecular masses in kDa derived from the BenchMark Pre-stained molecular markers (Invitrogen, USA).

**Figure 2:**
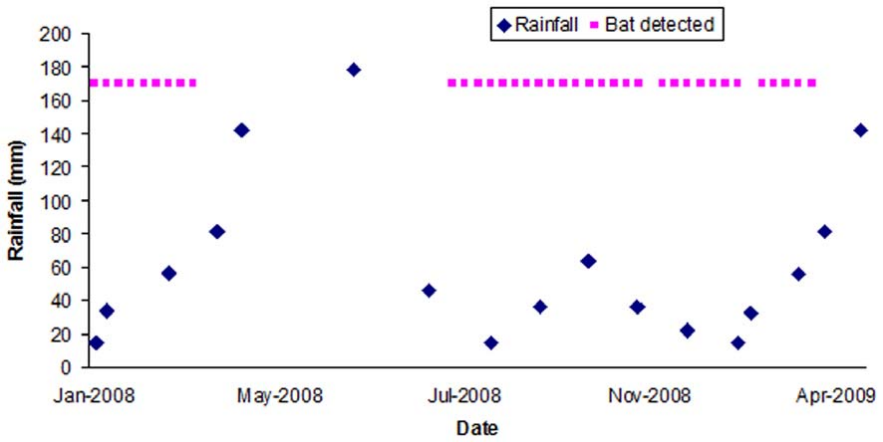
Weekly presence determined by radio-telemetry of the female *Eidolon helvum* in central Accra, Ghana. The bat was determined to be seropositive against both Ebolavirus subtype Zaire and Lagos Bat Virus (Nigeria 1956 isolate). The bat was pregnant when sampled in January 2008. Monthly mean rainfall is shown in mm (data from World Weather Information Service).

One pregnant adult female *E. helvum* (#49), sampled and released in Accra in January 2008, had an IgG antibody titer against EBOV of >1∶80, but was seronegative for MARV. All other samples were seronegative to both MARV and EBOV. The positive reactivity for EBOV was confirmed using western blot against a recombinant nucleocapsid protein of EBOV-Zaire, which was cloned and produced in an *E. coli* expression vector with His tag [13]. A total of 20 µg purified protein was separated in a preparative gel, followed by blotting and preparation of membrane strips (each containing 1.2–1.4 µg protein). Out of five bat sera tested (Figure 1), only sample #49 showed a clear and strong reactivity at a serum dilution of 1∶100. This pregnant bat also had a neutralizing antibody titer of >1∶80 against the 1956 Nigerian *Eidolon helvum* lyssavirus, Lagos bat virus (LBV), but no antibodies against Mokola virus using the 1968 Nigerian shrew (*Crocidura* sp.) isolate [9]. These bats were part of a large capture-mark-recapture project monitoring antibody seroprevalence to a range of pathogens and therefore no tissues were collected and insufficient heat-treated sera were available for RT-PCR. The EBOV-seropositive bat had been fitted with a radio transmitter and was last detected (using a SIKA Radio Tracking Receiver, BioTrack, Dorset, UK) in Accra in March 2009 (Figure 2) after which the colony migrated for the second time during the study. Weekly efforts were made to detect it until August 2009, but ceased due to the estimated battery life of the collar being 491 days. Therefore the animal will not be detected again even if it survives and returns to Accra from migration.

## Discussion

The novel finding in this study was that an individual female *E. helvum*, which had specific antibodies to both EBOV-Zaire and LBV, was alive over 13 months post-sampling. This bat appeared to be healthy and showed typical migratory movements for *E. helvum* in this region of West Africa [8] (Figure 2). Athough fruit bats seropositive to EBOV and MARV have been detected elsewhere, antibodies to filoviruses have not been previously demonstrated in *E. helvum*, including *E. helvum* sympatric with *R. aegyptiacus* seropositive to EBOV and MARV [2], and long-term survival of seropositive bats has not previously been shown.

Fruit bats are recognised reservoirs of lyssaviruses and henipaviruses, and can have high viral seroprevalences [9], [10], [14]. In contrast, most studies have reported that filovirus seroprevalence in bats is low (<10%) [1], [2], [3], [4], [15], although 24% *H. monstrosus* were found to be positive against EBOV following an EBOV epidemic in humans, and where PCR-positive bats were detected in the region shortly after the outbreak, suggesting recent virus circulation [4]. The positive predictive values of tests used in all of these studies are unknown. In the absence of a population of *E. helvum* known to be uninfected, formal investigation of specificity in our study is impossible. Similarly, the sensitivities of filovirus serological assays on bat samples are unknown. Efforts to ensure that results are accurate here were based on good laboratory approach and the use of western blot analysis to confirm IFA results.

One in 262 sera is clearly a low seroprevalence and it is likely that virus prevalence is extremely low in *E. helvum* in Ghana. Prevalences of other bat viruses, such as lyssaviruses and henipaviruses, for example, are very low even when seroprevalences are relatively high (e.g. around 40%) [14], [16], [17], [18], [19], [20], [21]. Western blot analysis indicated that the antibody response in our study was specific to EBOV-Zaire, which is highly pathogenic to people, rather than to EBOV-Reston (data not shown), which has low pathogenicity in humans and which has not been detected in Africa [22]. Whilst the pathogenicity of EBOV-Zaire to *E. helvum* cannot be determined by our study, the presence of detectable anti-EBOV antibody demonstrates that there has been sufficient exposure to viral antigen for the bat (#49) to develop an adaptive immune response. The most parsimonious explanation is infection followed by sufficient virus replication for the animal to mount an adaptive immune response. Insufficient data exist to determine if infection of *E. helvum* with EBOV can be productive, thus enabling it to serve as an effective reservoir for EBOV, or if this species develops clinical signs when infected with EBOV. Also, studies in individual bat roosts should not be extrapolated to all bats or roosts of that species, but population level infection dynamics must be considered when determining the reservoir status of a species.

Spillover of infection into a large susceptible population, such as *E. helvum*, which roosts in urban areas, must be considered a possible public health risk. Because *E. helvum* typically lives in large urban colonies and is a source of bushmeat in some regions [9] and direct bat to human transmission of EBOV has been reported [5], further studies should determine if this species forms a reservoir for EBOV from which spillover infections into the human population may occur.
